# The need for antibiotic stewardship and treatment standardization in the care of cirrhotic patients with spontaneous bacterial peritonitis – a retrospective cohort study examining the effect of ceftriaxone dosing

**DOI:** 10.12688/f1000research.3-57.v2

**Published:** 2014-07-14

**Authors:** Laura Mazer, Elliot B. Tapper, Gail Piatkowski, Michelle Lai

**Affiliations:** 1 Department of Surgery, Beth Israel Deaconess Medical Centre, Boston, MA, 02215, USA; 2 Division of Gastroenterology, Beth Israel Deaconess Medical Centre, Boston, MA, 02215, USA; 3 Decision Support, Beth Israel Deaconess Medical Centre, Boston, MA, 02215, USA

## Abstract

**Background:** Spontaneous bacterial peritonitis (SBP) is a common, often fatal affliction for cirrhotic patients. Despite all clinical trials of ceftriaxone for SBP using 2g daily, it is often given at 1g daily.

**Aim:** We evaluated survival after SBP as a function of ceftriaxone dosage.

**Methods:**  A retrospective cohort of all patients who received ceftriaxone for SBP (greater than 250 neutrophils in the ascites).

**Results:** As opposed to 1 gram, median survival is longer for patients receiving 2 grams (228 days vs. 102 days (p = 0.26) and one year survival is significantly higher (p = 0.0034).  After adjusting for baseline Model for End Stage Liver Disease (MELD) score, however, this difference was no longer significant.  Similarly, there was a significantly shorter length of intensive care for patients receiving 2 g (0.59 ± 1.78 days vs. 3.26 ± 6.9, p = 0.034), odds ratio 0.11 (95% CI 0.02 - 0.65). This difference, too, was no longer significant after controlling for the MELD score - odds ratio 0.21 (95% CI 0.04 - 1.07). Additionally, 70% of patients received at least one additional antibiotic; over 25 different medications were used in various combinations.

**Conclusions:**  Patients receiving 2 g of ceftriaxone may require fewer intensive care days and may enjoy an improved survival compared to those receiving 1 g daily. The complexity of antibiotic regimens to which cirrhotic patients are exposed must be studied further and rationalized.  We recommend fastidious antibiotic stewardship for patients with cirrhosis. Efforts should be made to craft local standards for the treatment of SBP that include appropriate antibiotic selection and dose.

## Introduction

Ascites is the most common hepatic decompensation, occurring in 50% of cirrhotic patients followed for over a decade ^1^. The development of ascites heralds a vulnerable time of sharply increased mortality for patients with liver disease – related in large part to spontaneous bacterial peritonitis (SBP) ^2, 3^. SBP is an infection of the ascitic fluid that occurs in 10–30% of patients with ascites ^4^. Fatal in as many as 32.6% of cases, SBP can have a profound effect on the tenuous hemodynamics of patients with cirrhosis ^5^. Exacerbating the arterial underfilling resulting from the splanchnic vasodilation of cirrhosis, SBP may lead to a decrease in cardiac output such that it can no longer satisfy the needs of a kidney that is already vasoconstricted ^6^. The result is the hepatorenal syndrome which is often devastating. SBP with renal injury is fatal in 42% of patients ^7^.

SBP is caused by translocation of gastrointestinal organisms into the ascitic fluid, most commonly *Escherichia coli*, *Klebsiella pneumoniae* and *Streptococcus pneumoniae*. As such, third generation cephalosporins are amongst the best studied antibiotics in this setting, with ceftriaxone as the drug of choice where cefotaxime is not available. Studied as a treatment for SBP in clinical trials for 25 years, the doses employed have been either 1 g every 12 hours or 2 g every 24 hours given intravenously for 5 to 10 day courses ^8– 14^.

At our center, we have found that ceftriaxone is often given at 1 g daily either in reference to online resources from other major teaching institutions or because 1 g is the general preset dose for this antibiotic as generated by the electronic ordering system ^15^. ( http://clinicalpharmacy.ucsf.edu/idmp/adult_guide/empiric_guide/intraabd_hosp_frame.htm, last accessed 1-12-2014). The outcomes of SBP as a function of ceftriaxone dosage – 1 g daily versus 2 g daily – have never been evaluated. It is unknown what effect the dosage of ceftriaxone has on the control of SBP or on mortality. Neither the American Association for the Study of Liver Disease (AASLD) nor the European Association for the Study of the Liver (EASL) guidelines on SBP management explicitly comment on the dosing of ceftriaxone for this indication ^16, 17^.

Herein we present the results of a retrospective review of the outcomes of SBP stratified by dose of ceftriaxone. This study aims to determine the difference in overall survival and intensive care utilization after an episode of SBP treated with differing doses of ceftriaxone.

## Methods

This is a retrospective, single center review of prospectively maintained medical records for all consecutive patients treated with ceftriaxone for SBP at the Beth Israel Deaconess Medical Center, Boston, USA, between January 2003 and December 2011.

We searched our clinical database for all patients that received ceftriaxone within 48 hours of a peritoneal fluid cell count and differential drawn in the emergency department or hospital ward. We then limited the population to those with 250 or more neutrophils in the ascites. Patient charts were then examined to exclude those with a prior liver transplant, evidence of intra-abdominal source of infection [abscess, perforation, recent (within 2 weeks) intra-abdominal surgery], peritoneal dialysis, ciprofloxacin or trimethroprim-sulfamethoxazole antibiotic prophylaxis, or documentation of a secondary infection (urinary tract infection, pneumonia, blood stream infection, cellulitis, meningitis) for which ceftriaxone was started prior to the peritoneal fluid collection. We collected data on age, sex, Model for End Stage Liver Disease (MELD) score (bilirubin, creatinine and PT/INR) at diagnosis, peritoneal white blood cell count and differential, blood and peritoneal culture data, dose of ceftriaxone, additional antibiotics, duration of antibiotic therapy, creatinine trends, intensive care utilization, length of hospital stay and mortality. Dates of death were confirmed in the medical record with reference to the Social Security Death Index. The cause of death was not collected as many patients died elsewhere. The primary outcome was overall survival after SBP diagnosis. Other outcomes included discharge creatinine, hospital length of stay and intensive care unit (ICU) length of stay.

Statistics were performed using SAS 9.2 and included student’s t-test, multivariate regression analysis, and log-rank testing/survival analysis where appropriate. P-value of 0.05 was considered significant for all analyses. While no prior studies have examined the effect of ceftriaxone dosing in order to determine study power, prior studies of ceftriaxone for SBP may be instructive. For example, in comparing 2 g ceftriaxone to cefonicid, the in-hospital death rate during therapy was 13% versus 30% which, assuming an alpha of 0.05, a sample size of 91 gives an 80% power ^13^. However, when examining the broader literature on ceftriaxone, regimens of variable duration (5 vs. 10 days) with 30% vs. 35% 30 day mortality would imply that studies require more than 1600 patients for adequate power ^8– 14^.

## Results

We found 138 patients with SBP treated with ceftriaxone. Of these, 91 patients met our inclusion criteria: 34 patients received 1 g daily and 57 received 2 g (total) daily. There was no significant difference between the groups with respect to age, gender, MELD score, peritoneal culture positivity or other infectious burden ( Table 1). All patients had received a protocol of albumin infusion on days 1 and 3 after the diagnosis of SBP in accordance with best practice ^18^.

**Table 1.** Patient characteristics for 1 g versus 2 g ceftriaxone dose, given as N (%) for categorical variables or mean ± SD for continuous variables.

	Ceftriaxone 1 g (N=34)	Ceftriaxone 2 g (N=57)	p-value
Patient age (years)	59.59 ± 11.24	55.10 ± 13.45	0.105
Female gender	9 (26%)	19 (33%)	0.527
MELD	20.55 ± 8.17	18.16 ± 6.48	0.125
Culture positive SBP	6 (18%)	6 (11%)	0.331
Other infectious source*	5 (14%)	9 (16%)	0.890

[[i] *Looking at patients admitted to a floor service, excluding prior transplants and prior episodes of SBP.*] [[ii] *Patients with documented pneumonia or urinary tract infection] [[iii] *MELD = Model for End-Stage Liver Disease. SBP = Spontaneous Bacterial Peritonitis.*]

**Figure 1.**
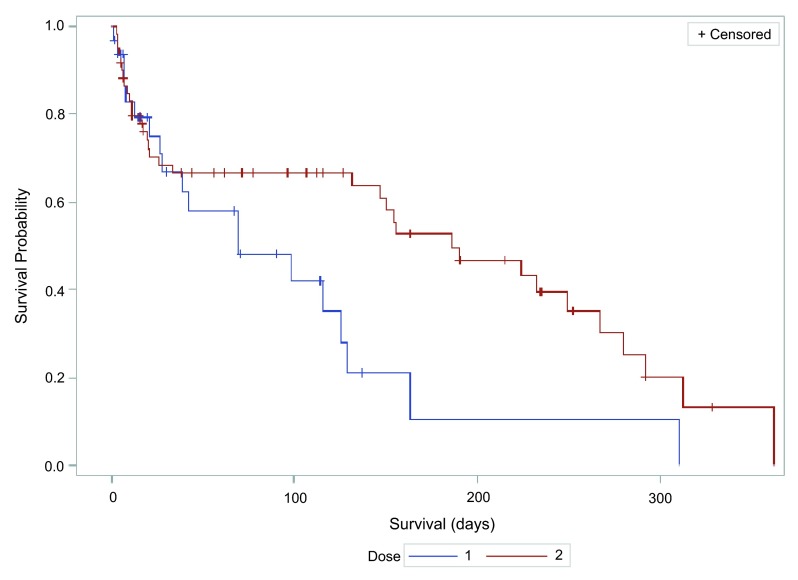
Kaplan-Meier survival curve after treatment of spontaneous bacterial peritonitis with 1 or 2 g ceftriaxone.

We next compared the hospital course for patients that received either dose of ceftriaxone ( Table 2). While both groups were likely to be treated with at least one additional antibiotic during their hospitalization (74% of those treated with 1 g, and 61% of those treated with 2 g), this difference was not significant. The total course of antibiotics – ceftriaxone or otherwise – was also similar between groups. The group receiving 2 g ceftriaxone daily did have a trend towards a shorter hospital stay, although this did not meet statistical significance (13.24 days vs. 10.28, p = 0.44). We did see a statistically significant shorter average length of intensive unit (ICU) stay in patients who received 2 g ceftriaxone a day (0.59 ± 1.78 days), compared to those who received 1 g ceftriaxone daily (3.26 ± 6.9 days) (p = 0.034). The odds ratio for ICU utilization was 0.11 (95% CI 0.02–0.65). However, this difference was no longer significant after controlling for MELD score - odds ratio 0.21 (95% CI 0.04–1.07). Finally, we examined one-year survival for patients treated with 1 versus 2 g ceftriaxone, and found a significant improvement in survival associated with the 2 g dose (p 0.0034 log rank test) ( Figure 1). Median overall survival was greater for patients treated with the 2 g dose (228 days vs. 102 days, however it was not significant (p = 0.26).

**Table 2.** Hospital course characteristics by ceftriaxone dose.

	1 g (N=34)	2 g (N=57)	p-value
Length of stay (days)	13.24 ± 21.5	10.28 ± 7.2	0.443
ICU days	3.26 ± 6.9	0.59 ± 1.78	0.034*
Repeat paracentesis at index hospitalization (N, %)	14 (41)	32 (56)	0.167
Repeat paracentesis with >250 neutrophils (N, %)	7 (21)	11 (19)	0.881
30-day readmission (N, %)	11 (32)	16 (28)	0.665
Other inpatient antibiotics (N, %)	25 (74)	35 (61)	0.238
Total inpatient antibiotic days	8.4 ± 8.5	8.8 ± 6.3	0.821
Inpatient duration of ceftriaxone (days)	4.8 ± 3.1	5.3 ± 3.2	0.491
Creatinine at discharge	1.57 ± 1.04	1.41 ± 1.46	0.529

[[i] ICU = intensive care unit. * Not significant after controlling for MELD score]

Given the high prevalence of additional antibiotic treatment, we also examined the pattern of antibiotic use. Overall, 70% of patients were treated with at least one additional antibiotic. The duration of antibiotic use, as well as the number and type of antibiotics prescribed were highly variable ( Table 3). While vancomycin was the most common concurrent antibiotic, used in 46% of patients, over 25 different medications were used in a variety of combinations. To further understand the antibiotic regimens observed, we next examined the available culture data. Of 91 patients diagnosed with SBP on neutrophil criteria, 13 were culture-positive. Of these, one patient had a documented infection resistant to ceftriaxone. This patient was excluded from the analysis. 14 patients had evidence of a secondary infection ( Table 1). These included pneumonia (diagnosed with chest x-ray), urinary tract infection (>100,000 colonies on urine dipstick with positive urine culture), and cellulitis (clinical diagnosis documented in chart).

**Table 3.** Types of inpatient antibiotics prescribed in addition to ceftriaxone, with number of patients and percentage of total population (n=138) and range of duration of inpatient antibiotic coverage (days).

Antibiotic	Number of patients N (%)	Duration range (days)
Vancomycin	63 (46)	1–22
Metronidazole	39 (28)	1–113
Piperacillin-tazobactam	26 (19)	1–23
Levofloxacin	21 (15)	1–43
Ciprofloxacin*	20 (15)	1–14
Cefepime	13 (9)	1–14
Meropenem	8 (6)	1–10
Ampicillin-sulbactam	6 (4)	1–37
Ceftazidime	6 (4)	1–9
Ampicillin	4 (3)	2–7
Azithromycin	4 (3)	1–6
Fluconazole	4 (3)	1–37
Nafcillin	3 (2)	2–6
Clindamycin	3 (2)	2–6
Gentamycin	2 (1)	6–9
Daptomycin	2 (1)	5–7
Amoxicillin	2 (1)	3–6
Trimethoprim- sulphamethoxazole*	1 (<1)	3
Ertapenem	1 (<1)	2
Clarithromycin	1 (<1)	3
Caspafungin	1 (<1)	11
Micafungin	1 (<1)	1
Aztreonam	1 (<1)	2
Moxifloxacin	1 (<1)	2
Linezolid	1 (<1)	9
Cefotaxime	1 (<1)	1
Cefazolin	1 (<1)	3

[[i] *Recorded as treatment. This analysis excluded patients continued on ciprofloxacin or trimethoprim-sulphamethoxazole for prophylaxis.]

Spontaneous bacterial peritonitis outcome and ceftriaxone dosage data Anonymized outcome data from medical records of patients treated with ceftriaxone for spontaneous bacterial peritonitis at the Beth Israel Deaconess Medical Center, Boston, USA, between January 2003 and December 2011. Exclusion criteria from dataset were: <250 neutrophils in ascites, prior liver transplant, evidence of intra-abdominal source of infection (abscess, perforation, recent (within 2 weeks) intra-abdominal surgery), peritoneal dialysis and documentation of a secondary infection (urinary tract infection, pneumonia, blood stream infection, cellulitis, meningitis) for which ceftriaxone was started prior to peritoneal fluid collection.

## Conclusion

Our study of ceftriaxone dosage for SBP yielded three core findings. First, there was a trend towards improved mortality with the 2 g dosage, however we did not detect a difference between the groups receiving either dose of ceftriaxone and the rate of in-hospital mortality or the rate of persistent renal injury. Second, a total ceftriaxone dose of 2 g daily over 1 g daily exhibited a non-significant reduction of intensive care utilization by cirrhotic patients with SBP, after adjusting for MELD score. Prospective studies in a larger cohort are indicated to explore the true significance of these results. While it could explain our results, whether the pharmacodynamics of intravenous ceftriaxone are such that the peritoneal drug concentration following a 1 g infusion results in slower control of infection is unclear from our study ^19^. Third, the number, duration and complexity of antibiotic regimens that cirrhotic patients experience is highly variable. The reasons for this finding are unclear and deserve further study in order to understand both the physician and patient factors that increase antibiotic regimen complexity as well as the effect on outcomes including mortality, morbidity and future infection with resistant organisms. Ultimately, we feel that the complexity of antibiotic regimens speaks to clinical uncertainty and the urgent need to improve the yield of ascitic cultures and tailor therapy for SBP with consideration of local microbiological data.

**Figure 2.**
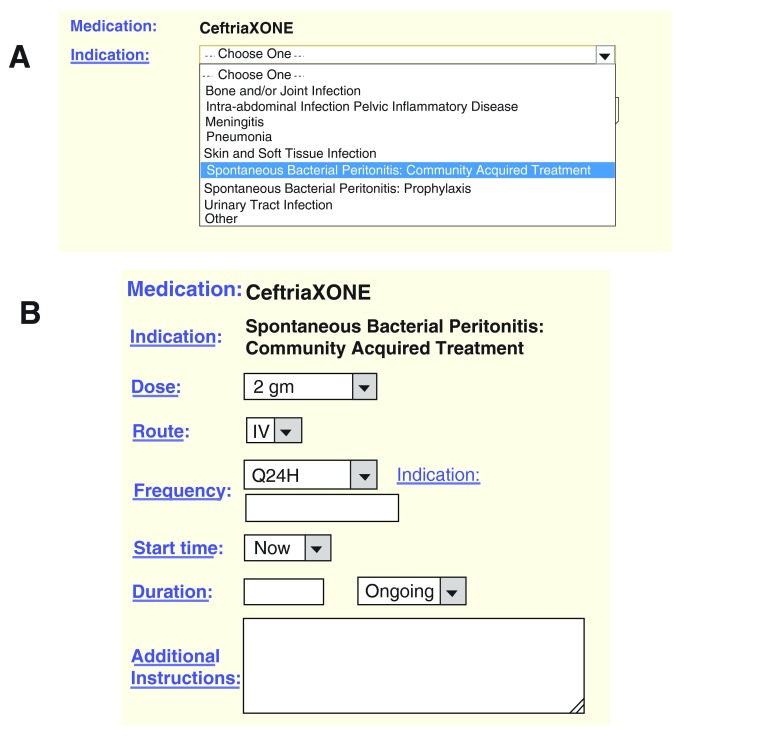
Modified provider order entry standardizes treatment of spontaneous bacterial peritonitis. **A**. When an ordering physician chooses ceftriaxone, an indication must be chosen. **B**. When spontaneous bacterial peritonitis is the chosen indication, the preset dose is 2 g daily.

This study emphasizes the need for antibiotic stewardship and treatment standardization in the care of cirrhotic patients. We feel this can be easily achieved by computer programming. For centers that use electronic provider order entry, a preset dose of 2 g of ceftriaxone when prescribing for a diagnosis of SBP can ensure standardized and appropriate dosing. Beyond that, we have programmed a prompt into ceftriaxone orders that asks the physician to specify whether the medication is intended to treat SBP. This selection results in an automatic 2 g daily dose ( Figure 2). This has two purposes. First, by selecting an indication for the antibiotic, we are effectively able to track our patients treated for SBP prospectively for quality assurance purposes. Second, while our findings are inconclusive, they are suggestive of a benefit from a higher ceftriaxone dose, which the study may have ultimately been underpowered to detect. Cirrhotic patients can be admitted to any service of the hospital, including those staffed by hepatologists, internists, surgeons and intensivists. By standardizing care delivery, the healthcare system can ensure that the medications cirrhotic patients receive are dosed appropriately for their needs. Furthermore, by programming a menu-selection for SBP, our hospital - or any hospital with similar capabilities - may track the disease indications for each antibiotic allowing for audits and outreach.

Our conclusions are limited in a few ways. First, our study is retrospective and therefore we cannot comment on the impact of other treatment decisions that may or may not be associated with the dose of ceftriaxone chosen. We can speak only to the association of ceftriaxone dose with mortality, not causation. Furthermore, given the fragmented nature of clinical care, it is impossible to know the cause of death in all patients. Additionally, we cannot exclude the possibility that our study was underpowered to detect a difference between treatment groups. Second, the microbiology of our patients’ SBP is unclear given the low rate of culture positivity so we cannot comment on the impact of antimicrobial resistance. Third, follow-up paracenteses to confirm resolution of the SBP after antibiotic treatment were infrequent and thus we cannot comment on the rate of resolution of neutrophilia as function of ceftriaxone dose.

In order to prevent unwanted practice variation, we recommend standardizing the treatment of SBP by automating the dose of ceftriaxone in the provider order entry system. Further research must be aimed at rationalizing the antibiotic regimens employed in the treatment of cirrhotic patients. Programs to this end include fastidious antibiotic stewardship facilitated by computerized audits of indication-based antibiotic usage and improved microbial culture and detection techniques.

## Data availability

figshare: Spontaneous bacterial peritonitis outcome and ceftriaxone dosage data, doi: http://dx.doi.org/10.6084/m9.figshare.931754 ^20^
