# Supplementary material for: The need for antibiotic stewardship and treatment standardization in the care of cirrhotic patients with spontaneous bacterial peritonitis – a retrospective cohort study examining the effect of ceftriaxone dosing
Source: F1000Res. 2014 Jul 14;3:57. Originally published 2014 Feb 14. [Version 2] doi: 10.12688/f1000research.3-57.v2 (PMC4133760; doi:10.12688/f1000research.3-57.v2)
Supplement: Peer review history

# Peer review: Referee response for version 2

**DOI:** 10.5256/f1000research.4910.r5502

**Published:** 2014-07-21

**Authors:** Andres Cardenas

**Affiliations:** 1 Institut Clinic de Malalties Digestives i Metaboliques, Hospital Clinic, University of Barcelona, Barcelona, Spain

The article now looks good and I do not have any further comments.

I have read this submission. I believe that I have an appropriate level of expertise to confirm that it is of an acceptable scientific standard.

# Peer review: Referee response for version 1

**DOI:** 10.5256/f1000research.3447.r3998

**Published:** 2014-04-10

**Authors:** Andres Cardenas

**Affiliations:** 1 Institut Clinic de Malalties Digestives i Metaboliques, Hospital Clinic, University of Barcelona, Barcelona, Spain

This is an interesting retrospective study where the authors review the effectiveness of ceftriaxone dosing for patients with SBP. I like the article and the principles behind the analysis.

**Major points:**

1. The aim of the study should be better explained - what did the authors set out to study? Cure, outcomes, prognosis?
2. The title should reflect the retrospective nature of the analysis.
3. Were any patients on antibiotic prophylaxis (i.e norfloxacion or cipro) prior to the dx of SBP or other bacterial infections?
4. There is a trend of differences in mortality. Why is this so - do the authors have the causes of death in both groups?

 

The authors’ message should be that there seems to be a difference and that this analysis paves the way for future randomized studies that take local microbiological data from each institution into account.

I have read this submission. I believe that I have an appropriate level of expertise to confirm that it is of an acceptable scientific standard, however I have significant reservations, as outlined above.

# Response

**Published:** 2014-06-18

**Authors:** Elliot Tapper

**Affiliations:** Department of Medicine, Beth Israel Deaconess Medical Center, USA

We appreciate Dr. Cardenas' comments. As regards to his major points, the first three were easily addressed. First, the title, abstract and introduction have been changed substantially to clarify the aims with the principle focus being patient outcomes. Second, the title has been changed. Third, we clarified that patients on antibiotic prophylaxis were excluded.

As for his fourth, unfortunately it is extremely difficult to determine causes of death when patients often die at other institutions. We do not feel that this is a major limitation for three main reasons. First, we confirm that the patients are alive or dead using a national database. Second, the available prognostics in liver disease, namely the MELD, are capable of predicting all-cause mortality and we adjust for MELD. Third, the causes of death in decompensated cirrhosis are fairly circumscribed and typically closely related to the patients’ antecedent clinical course. SBP can result in mortality via sepsis, and renal failure but also, potentially, variceal bleeding, if say the clinician held beta-blockade to preserve renal function.

# Peer review: Referee response for version 1

**DOI:** 10.5256/f1000research.3447.r4389

**Published:** 2014-04-08

**Authors:** Manuela Merli

**Affiliations:** 1 Department of Clinical Medicine, Sapienza University of Rome, Rome, Italy

This is a retrospective study aimed at evaluating the relationship between dosing of ceftriaxone (1 or 2 grams) and outcome in SBP.

This issue is certainly of interest however, as the authors stated in the discussion, the study is inconclusive due to several limitations which derive from the retrospective approach. For this reason I would also suggest that the title should be changed to underline the point that the main finding in the study is that uneventfully these patients may receive different antibiotic dosages for the same indication. A possible title could be “ *Need for antibiotic stewardship and treatment standardization in the care of cirrhotic patients*”

The answer to the question “ *should we use 1 or 2g ceftriaxone*?” can only be derived from a prospective randomized study. On the other hand it is unlikely that one year mortality (Figure 1) could have been influenced by the treatment of the index episode. In fact these patients had a similar length of stay, a similar in hospital mortality and a similar rate of 30 days readmission. From this point of view, one could even derive that the therapy with 1g ceftriaxone was not inferior to 2g ceftriaxone. I suggest these observations be taken into account in the text.

I have read this submission. I believe that I have an appropriate level of expertise to confirm that it is of an acceptable scientific standard, however I have significant reservations, as outlined above.

# Response

**Published:** 2014-06-18

**Authors:** Elliot Tapper

**Affiliations:** Department of Medicine, Beth Israel Deaconess Medical Center, USA

The reviewers’ points are well taken and have been adopted in the manuscript. Language reinforcing the statistically equal efficacy of ceftriaxone doses has been inserted at critical points. The notion that 1g is non-inferior to 2g however cannot be included as the study design does not allow for claims regarding inferiority. Furthermore, we believe that though the power calculation based on prior works suggested our sample size was sufficient, it is likely the case that when comparing 1g to 2g, the study was underpowered to confirm the trends toward improved outcomes with 2g.

# Peer review: Referee response for version 1

**DOI:** 10.5256/f1000research.3447.r3731

**Published:** 2014-02-25

**Authors:** José Castellote

**Affiliations:** 1 Gastroenterology Department, Hepatology Unit, Hospital Universitari de Bellvitge, Barcelona, Spain

This is an interesting retrospective study but I have the following comments:

1. The title is appropriate. I think that the abstract provides an adequate summary but the conclusions may need to be changed. The first sentence is a recommendation and the differences in one-year survival are difficult to explain purely on the basis of 7 days of antibiotic therapy.
2. In the method section the clinical endpoints that the authors are going to study should be explained and the following should be added: in-hospital mortality, infection cure rate, bacterial resistance and super-infections. Ascitic culture method should also be detailed.
3. What are the reasons for different mortality rates at one-year in both groups? This point is crucial and must be discussed in the discussion.
4. Lastly, I think that the study shows no differences in hospital mortality rate, infection control rate, renal failure, or hepatorenal syndrome between both groups and this should be pointed out in the conclusions.

I have read this submission. I believe that I have an appropriate level of expertise to confirm that it is of an acceptable scientific standard, however I have significant reservations, as outlined above.

# Response

**Published:** 2014-06-18

**Authors:** Elliot Tapper

**Affiliations:** Department of Medicine, Beth Israel Deaconess Medical Center, USA

The reviewer’s comments are well taken.

1. The language used in the conclusions has been moderated for less sweeping claims. The use of 1 year mortality after one clinical event is frequently described in the literature on cirrhosis. While SBP and 1 year mortality seem disconnected, SBP is a watershed moment for patients with ascites, the prognostic effects of which are well described.
2. Endpoints have been clarified. Mortality has been included. Infection cure rate is incompletely captured as very few patients received follow up paracentesis; patients with resistant species (very few) and super-infections were specifically excluded from the study. The ascitic culture method is standard.
3. We add a specific comment on the causes of mortality. Please see our response to Dr. Cardenas. We regret that we cannot provide actual causes of death. However, as the primary outcome was all-cause mortality and we controlled for factors that are validated to predict all-cause mortality, we feel that this outcome is legitimate.
4. Agreed.
